# Supplementary material for: Maternal antibodies facilitate Amyloid-β clearance by activating Fc-receptor-Syk-mediated phagocytosis
Source: Commun Biol. 2021 Mar 12;4:329. doi: 10.1038/s42003-021-01851-6 (PMC7955073; doi:10.1038/s42003-021-01851-6)
Supplement: Supplementary file 1 — Supplementary Information [file 42003_2021_1851_MOESM1_ESM.pdf]

Supplementary note

Effector *FcR* transcript levels negatively correlate with Aβ load in the brain. We assessed whether upregulation of FcRs was correlated with a reduction in Aβ load. *FcγRI*, *FcγRIII*, *FcγRIV*, *FcRn* and *FcγRIIb* mRNA levels negatively correlated with cortical SDS-soluble $A\beta_{40}$ levels (r=-0.64, P=0.001, r=-0.63, P=0.001, r=-0.43, P=0.02, r=-0.59, P=0.002, r=-0.56, P=0.004, respectively, Fig. S4a, left panel). Levels of TBST-soluble $A\beta_{42}$ negatively correlated with mRNA levels of *FcγRI* and *FcγRIII* (r=-0.43, P=0.02, r=-0.4, P=0.03, respectively, Fig. S4a, right panel) but not with *FcγRIV*, *FcRn*, or *FcγRIIb*. Additionally, *FcγRI*, *FcγRIII*, *FcγRIV*, *FcRn* and *FcγRIIb* mRNA levels negatively correlated with cortical SDS-soluble $A\beta_{42}$ levels (r=-0.57, P=0.004, r=-0.53, P=0.007, r=-0.51, P=0.009, r=-0.6, P=0.002, r=0.44, P=0.03, respectively, Fig. S4a, right panel). Importantly, *FcγRI*, *FcγRIII,* and *FcRn* were negatively correlated with insoluble $A\beta_{42}$ (r=-0.42, P=0.03, r=-0.4, P=0.03, r=-0.42, P=0.02, Fig. S4a, right panel, S4b, c, e). *FcγRIV* and *FcγRIIb* did not correlate with insoluble $A\beta_{42}$ (Fig. S4a, right panel, S4d, f).

Maternal vaccination elevates FcγRI on microglial cells. Ab specificity for these receptors was verified on mouse spleen sections (Fig. S5). Of these receptors, FcγRI was the only receptor found to be expressed in the brain (Fig. S6). Co-immunostaining with the cell-specific Iba1, NeuN, and GFAP markers revealed that FcγRI was exclusively expressed on Iba^+ microglia, but not $Neun^+$ neurons or $GFAP^+$ astrocytes (P<0.0001, Fig. S6a-b). The distribution of cellular expression on Iba^+, but not on $NeuN^+$ and $GFAP^+$, appears to be close to normal (mean=33.01, median=32.32, Fig. S6c, d), with no significant difference from a simulated normal distribution with the same mean and standard deviation (P=0.26, Fig S6c). FcγRI was thus expressed mainly on the vast majority of microglial cells, with its expression intensity normally distributed (Fig. S6c, d).

FcγRI is elevated in cortical microglia of maternally vaccinated mice. As seen in the hippocampus, cortical FcγRI levels were elevated in maternally vaccinated mice M+/A- and M+/A+ compared with both unvaccinated M-/A- and actively vaccinated mice M-/A+ ($P<0.01$, Fig. S7a-b). FcγRI distribution is normal in all vaccinated groups ($P=0.09$, $P=0.2$, $P=0.14$, compared to a simulated normal distribution, respectively, Fig. S7a, c), while this distribution in unvaccinated mice appears to be skewed ($P<0.05$, Fig. S7a, c). Active vaccination alone seems to elevate levels of microglial FcγRI compared with unvaccinated mice. This elevation did not reach significance in two-way ANOVA, although the distributions of the two groups did significantly differ ($P<0.0001$, Fig. S7d).

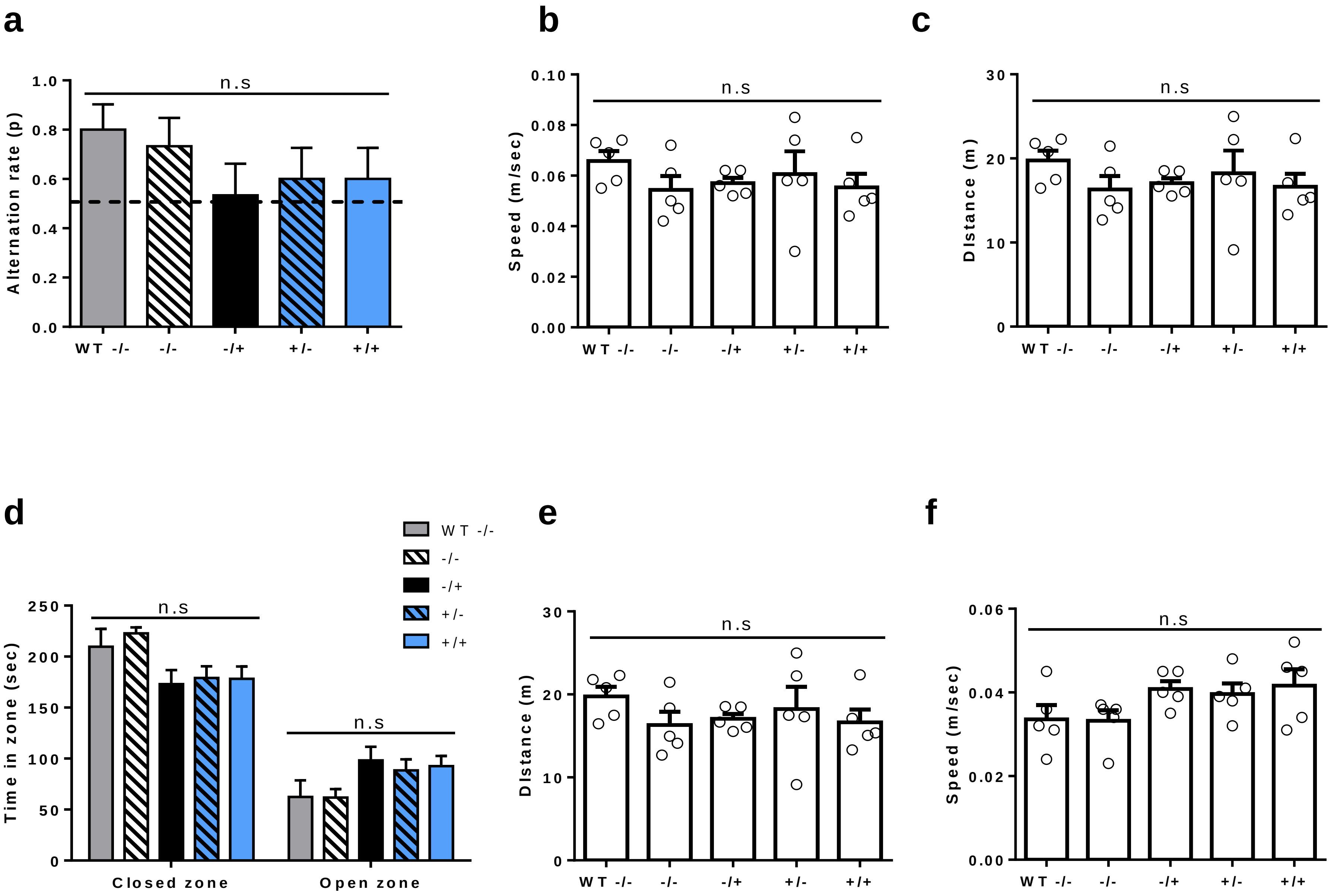
a
n.s
Alternation rate (p)
1.0
0.8
0.6
0.4
0.2
0.0
WT -/-
-/-
-/+
+/-
+/+
b
n.s
Speed (m/sec)
0.10
0.08
0.06
0.04
0.02
0.00
c
n.s
DIstance (m)
30
20
10
0
d
WT -/-
-/-
-/+
+/-
+/+
n.s
n.s
Time in zone (sec)
250
200
150
100
50
0
Closed zone
Open zone
e
n.s
DIstance (m)
f
n.s
Speed (m/sec)
0.06
0.04
0.02
0.00

Fig. S1

Figure S1. A combination of maternal and active vaccination rescues short-term memory abilities and normalizes exploratory behavior. Supporting data for the main behavioral figure. (a) Spontaneous alteration T-maze revealed no difference between groups (b) Speed and (c) distance traveled in the OF test were unchanged between groups, (d) Time spent in the open and close sections of the elevated zero-maze did not differ between groups, suggesting no differential effect of anxiety. (e) Distance and (f) speed in the EZM did not differ between groups. One-way ANOVA, two-way ANOVA, data is presented as mean±SEM.

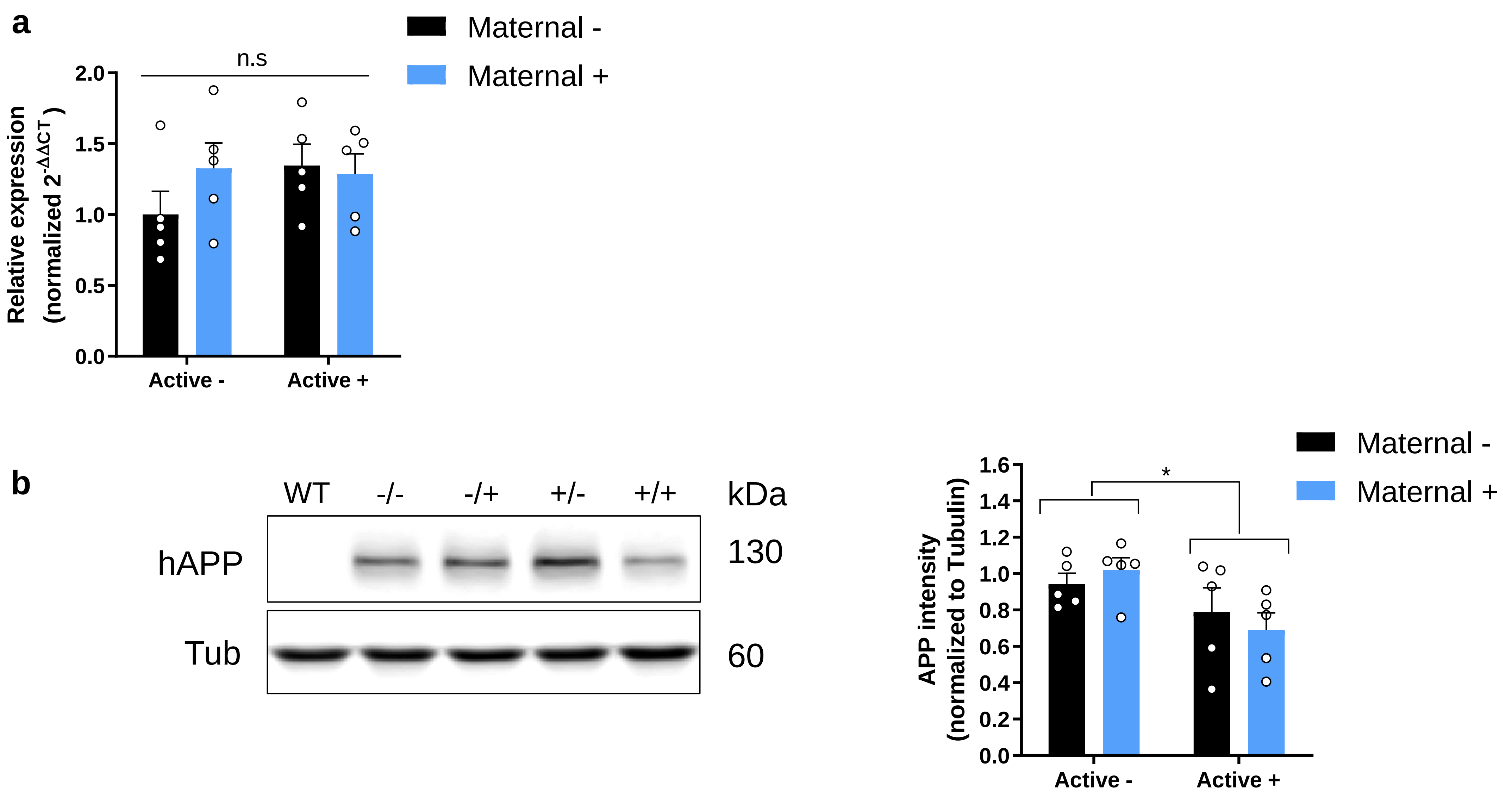
a
Maternal -
Maternal +
n.s
Relative expression
(normalized 2-ΔΔCT)
2.0
1.5
1.0
0.5
0.0
Active -
Active +
b
WT
-/-
-/+
+/-
+/+
kDa
hAPP
130
Tub
60
APP intensity
(normalized to Tubulin)
1.6
1.4
1.2
1.0
0.8
0.6
0.4
0.2
0.0
*
Active -
Active +
Maternal -
Maternal +

Fig. S2

Figure S2. Change in expression levels of hAPP does not account for cerebral Aβ levels following maternal and active vaccination. (a) Transcript levels of cortical *hAPP* do not differ between groups. (b) Active vaccination reduces cerebral hAPP levels alongside with reducing Aβ. *$P<0.05$, two-way ANOVA, data is presented as mean±SEM.

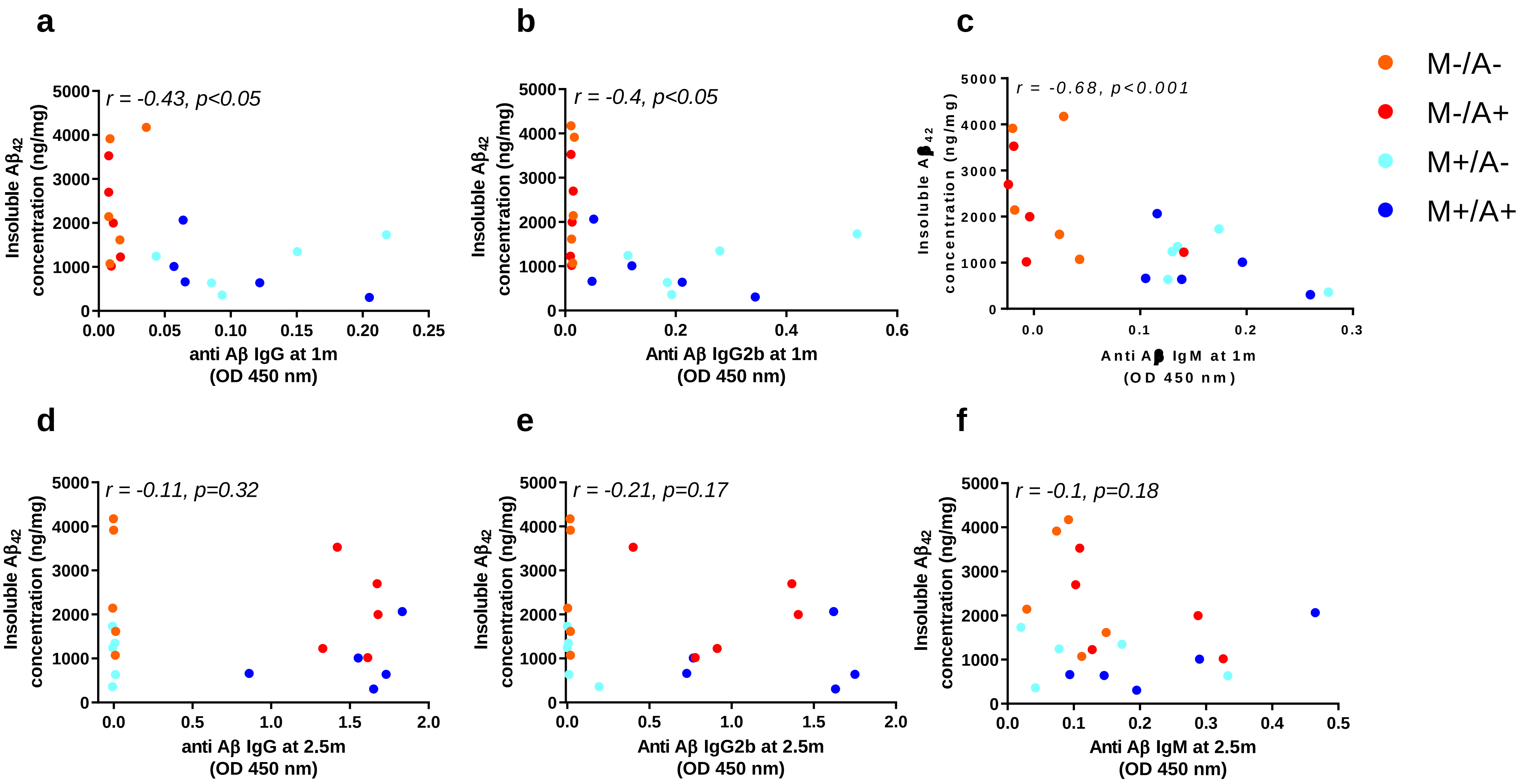

a
r = -0.43, p<0.05
Insoluble Aβ42 concentration (ng/mg)
anti Aβ IgG at 1m (OD 450 nm)
b
r = -0.4, p<0.05
Anti Aβ IgG2b at 1m (OD 450 nm)
c
r = -0.68, p<0.001
Anti Aβ IgM at 1m (OD 450 nm)
M-/A-
M-/A+
M+/A-
M+/A+
d
r = -0.11, p=0.32
anti Aβ IgG at 2.5m (OD 450 nm)
e
r = -0.21, p=0.17
Anti Aβ IgG2b at 2.5m (OD 450 nm)
f
r = -0.1, p=0.18
Anti Aβ IgM at 2.5m (OD 450 nm)

Fig. S3

Figure S3. Maternal, but not active vaccination predicts reduced Aβ pathology at adulthood. Scatter plot of insoluble $A\beta_{42}$ levels and IgG isotypes following maternal (a-c) and active (d-f) vaccination. (a) Total IgG, (b) IgG2b, and (c) IgM at 1m of age negatively correlate with Aβ pathology at adulthood. Levels of (d) total IgG, (e) IgG2b, and (f) IgM at 5m of age poorly correlate with Aβ pathology, Pearson's correlation.

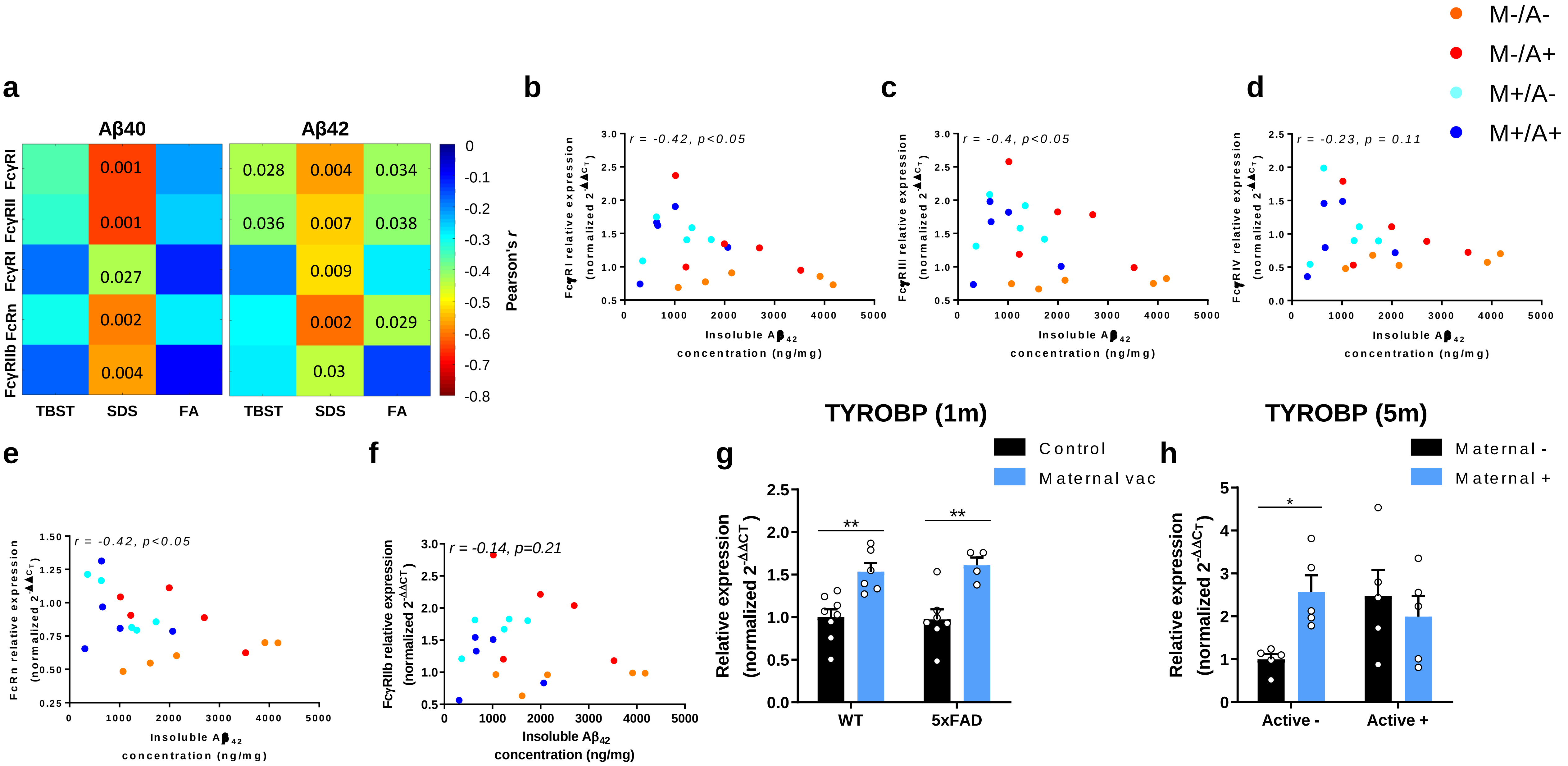

M-/A-
M-/A+
M+/A-
M+/A+
a
Aβ40
Aβ42
FcγRI
FcγRII
FcγRI
FcRn
FcγRIIb
0.001
0.001
0.027
0.002
0.004
0.028
0.004
0.034
0.036
0.007
0.038
0.009
0.002
0.029
0.03
TBST
SDS
FA
Pearson's r
b
r = -0.42, p<0.05
c
r = -0.4, p<0.05
d
r = -0.23, p = 0.11
Insoluble Aβ42 concentration (ng/mg)
TYROBP (1m)
TYROBP (5m)
e
r = -0.42, p<0.05
FcRn relative expression (normalized 2-ΔΔCT)
f
r = -0.14, p=0.21
FcγRIIb relative expression (normalized 2-ΔΔCT)
g
Control
Maternal vac
Relative expression (normalized 2-ΔΔCT)
WT
5xFAD
h
Maternal -
Maternal +
Active -
Active +

Fig. S4

Figure S4. Cerebral *FcγR* levels negatively correlate with Aβ pathology. (a) Levels of *FcγR*s at 5m of age negatively correlate with cerebral Aβ pathology. (b-f) Scatter plot of insoluble $A\beta_{42}$ levels and FcR expression levels at 5m of age present negative correlations. (b) *FcγRI*, (c) *FcγRIII,* (d) *FcγRIV,* (e) *FcRn*, and (f) *FcγRIIb*. (g) Cerebral levels of *TYROBP* following maternal vaccination at 1m of age (h) Cerebral levels of *TYROBP* following maternal and active vaccination at 5m of age. *P<0.05, **P<0.01, Pearson's correlation, Two-way ANOVA, data is presented as mean±SEM.

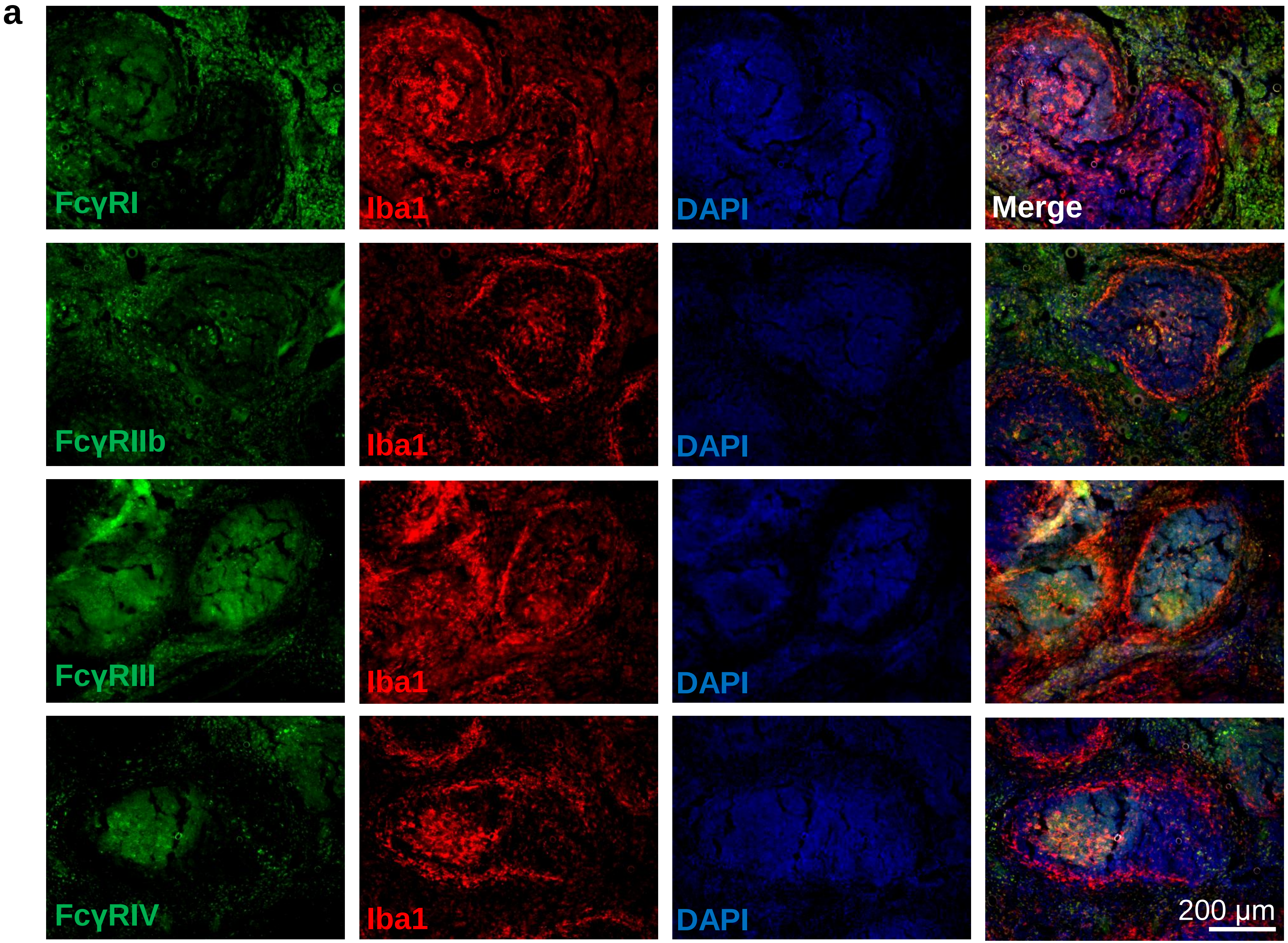
a
FcγRI
Iba1
DAPI
Merge
FcγRIIb
Iba1
DAPI
FcγRIII
Iba1
DAPI
FcγRIV
Iba1
DAPI
200 μm

Fig. S5

Figure S5. FcγRs are expressed in extra-follicular areas, the follicles and germinal centers in the spleen. FcR Ab specificity was verified in spleen tissue slices prior to use on brain sections. (a) FcγRI, FcγRIII, FcγRIV, and FcRn were double-labeled with Iba1$^+$ macrophages in the spleen. FcγRI is expressed on Iba1$^+$ cells located at the surroundings of splenic follicles, and FcγRIIb, FcγRIII, and FcγRIV are co-localized with cells at extra-follicular areas, the follicles, and germinal centers.

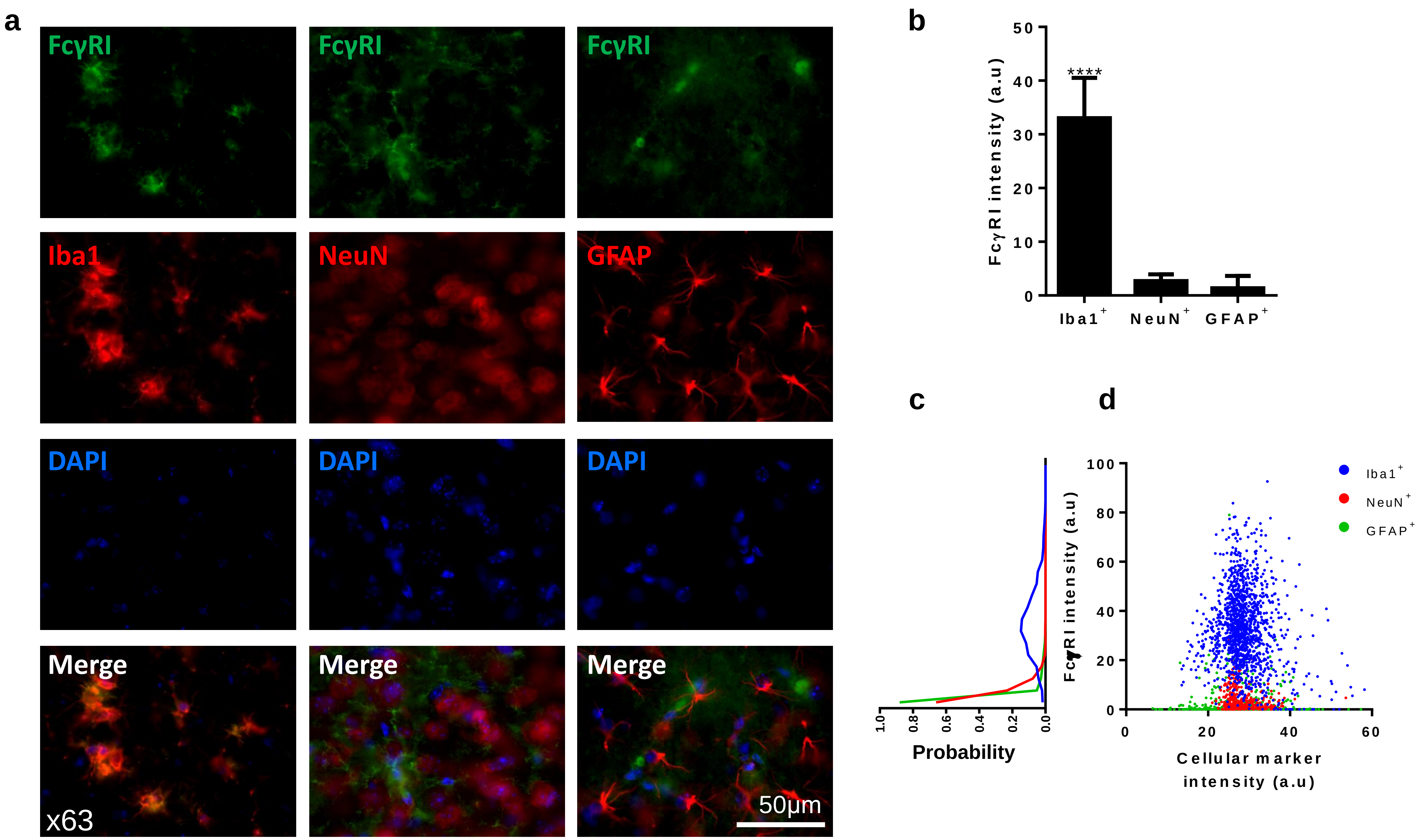
a
FcγRI
FcγRI
FcγRI
Iba1
NeuN
GFAP
DAPI
DAPI
DAPI
Merge
Merge
Merge
x63
50µm
b
FcγRI intensity (a.u)
****
Iba1+
NeuN+
GFAP+
c
Probability
d
FcγRI intensity (a.u)
Cellular marker intensity (a.u)
Iba1+
NeuN+
GFAP+

Fig. S6

Figure S6. FcγRI is expressed on microglial cells. To assess FcγRI expression among CNS cells, double labeling was conducted with (a) $Iba1^+$ (microglia), NeuN (mature neurons) and GFAP (astrocytes), using the x10 objective. (b) FcγRI fluorescent signal intensity is higher in $Iba1^+$ cells compared with both NeuN and $GFAP^+$ cells. (c) Scatter plot and distributions of FcγRI expression on microglia, neurons, and astrocytes, showing normal distribution among microglia and right-skewed distributions for astrocytes and neurons (d) FcγRI is expressed within the somas of Iba1 $^+$cells. Images were taken using the x40 (left panels) and x63 (right panels) objectives. ****$P<0.0001$, one-way ANOVA, data is presented as mean±SEM.

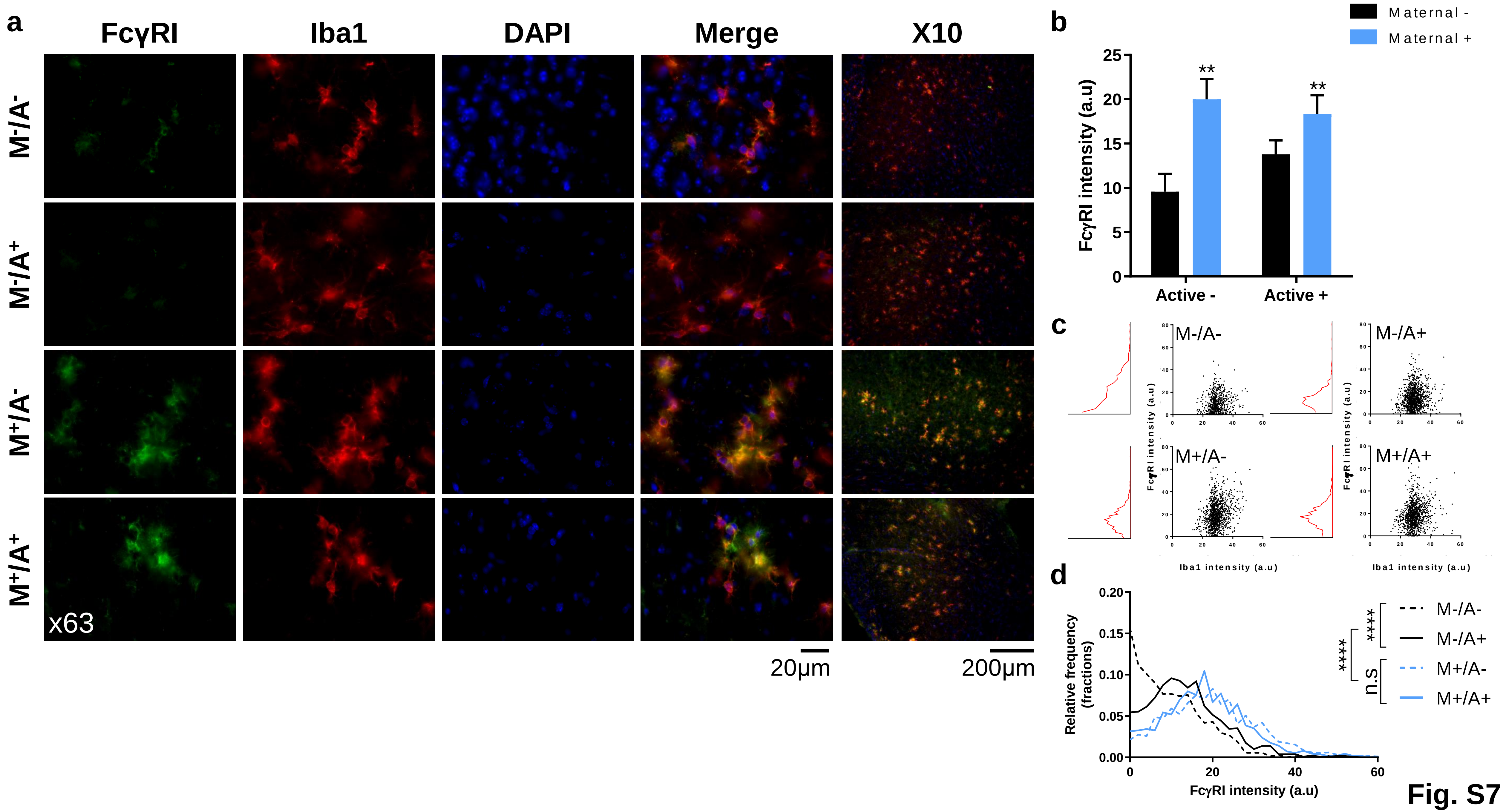
a
FcγRI
Iba1
DAPI
Merge
X10
M-/A-
M-/A+
M+/A-
M+/A+
x63
20µm
200µm
b
Maternal -
Maternal +
FcγRI intensity (a.u)
**
**
Active -
Active +
c
M-/A-
M-/A+
M+/A-
M+/A+
FcγRI intensity (a.u)
Iba1 intensity (a.u)
d
Relative frequency (fractions)
FcγRI intensity (a.u)
****
****
n.s
M-/A-
M-/A+
M+/A-
M+/A+

Fig. S7

Figure S7. FcγRI is upregulated in cortical microglia of maternally vaccinated 5xFAD mice. (a) FcγRI expression was assessed using double-labeled immunofluorescence with Iba1$^+$ microglia, using the x10 and x63 objectives for visualizing and quantification, respectively. (b) FcγRI signal was increased among microglia from maternally vaccinated mice independently of active vaccination. (c) Scatter plot of Iba1 and FcγRI signals reveal right-skewed distribution for FcγRI expression among unvaccinated and actively vaccinated mice, and normal distribution for maternally vaccinated mice. (d) Overlay and comparisons of FcγRI expression distribution. **$P<0.01$, ****$P<0.0001$, two-way ANOVA, corrected two-sample Kolmogorov-Smirnov test, data is presented as mean±SEM.

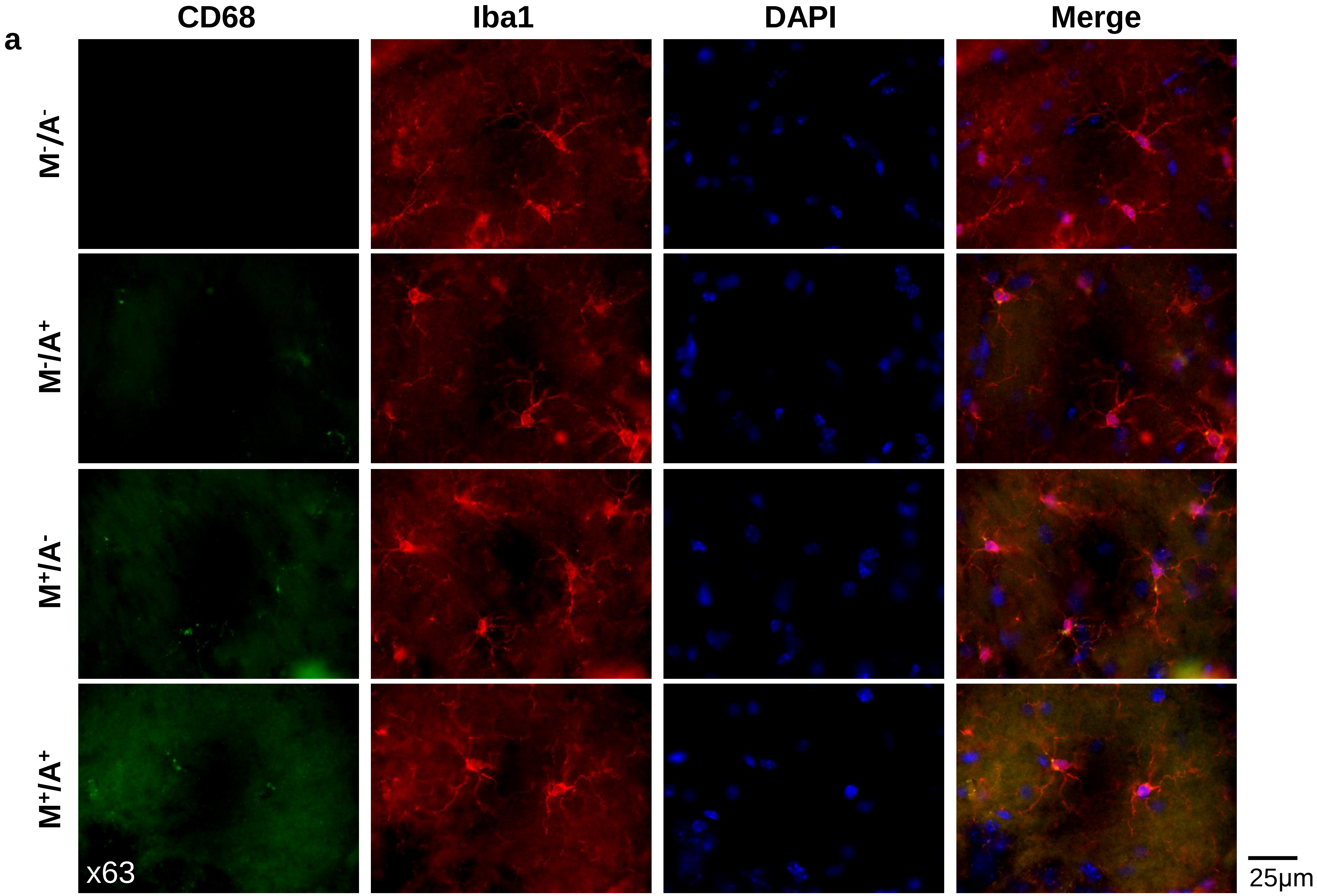

Fig. S8

Figure S8. CD68 expression following maternal and active vaccination in ramified homeostatic microglia. (a) Double labeling of CD68 and Iba1^{+} in ramified microglia, taken using the x63 objective.

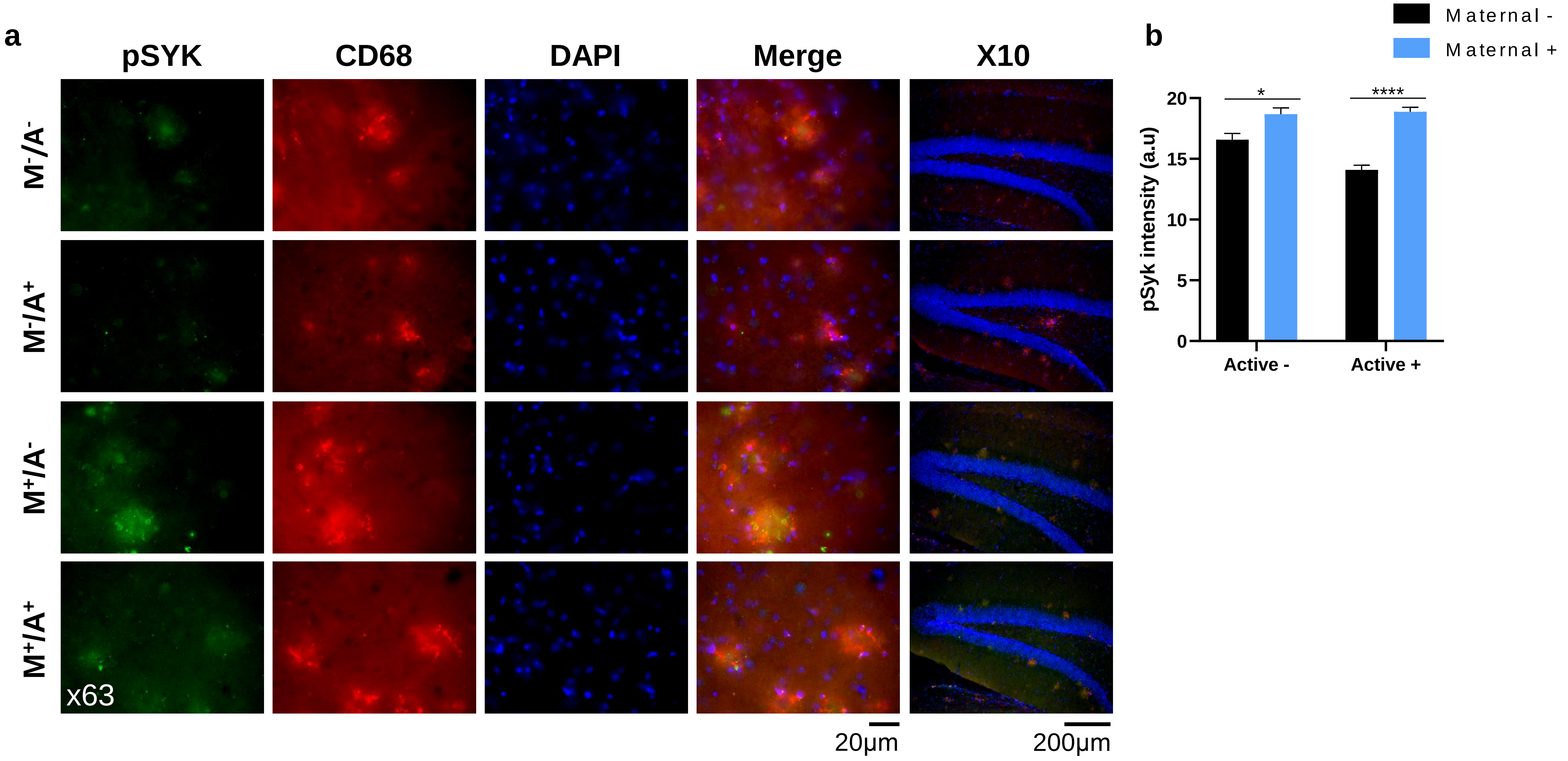
a
pSYK
CD68
DAPI
Merge
X10
M-/A-
M-/A+
M+/A-
M+/A+
x63
20µm
200µm
b
Maternal -
Maternal +
pSyk intensity (a.u)
20
15
10
5
0
*
****
Active -
Active +

Fig. S9

Figure S9. Maternal vaccination increases Syk activation in hippocampal microglia. (a) Syk activation among microglial cells was assessed using double labeling of pSyk and CD68 positive microglia, using the x10 and x63 objectives for visualizing and quantification, respectively. (b) pSyk signal was higher in maternally and actively immunized mice compared with unvaccinated controls. *P<0.05, ****P<0.0001, two-way ANOVA, data is presented as mean±SEM.

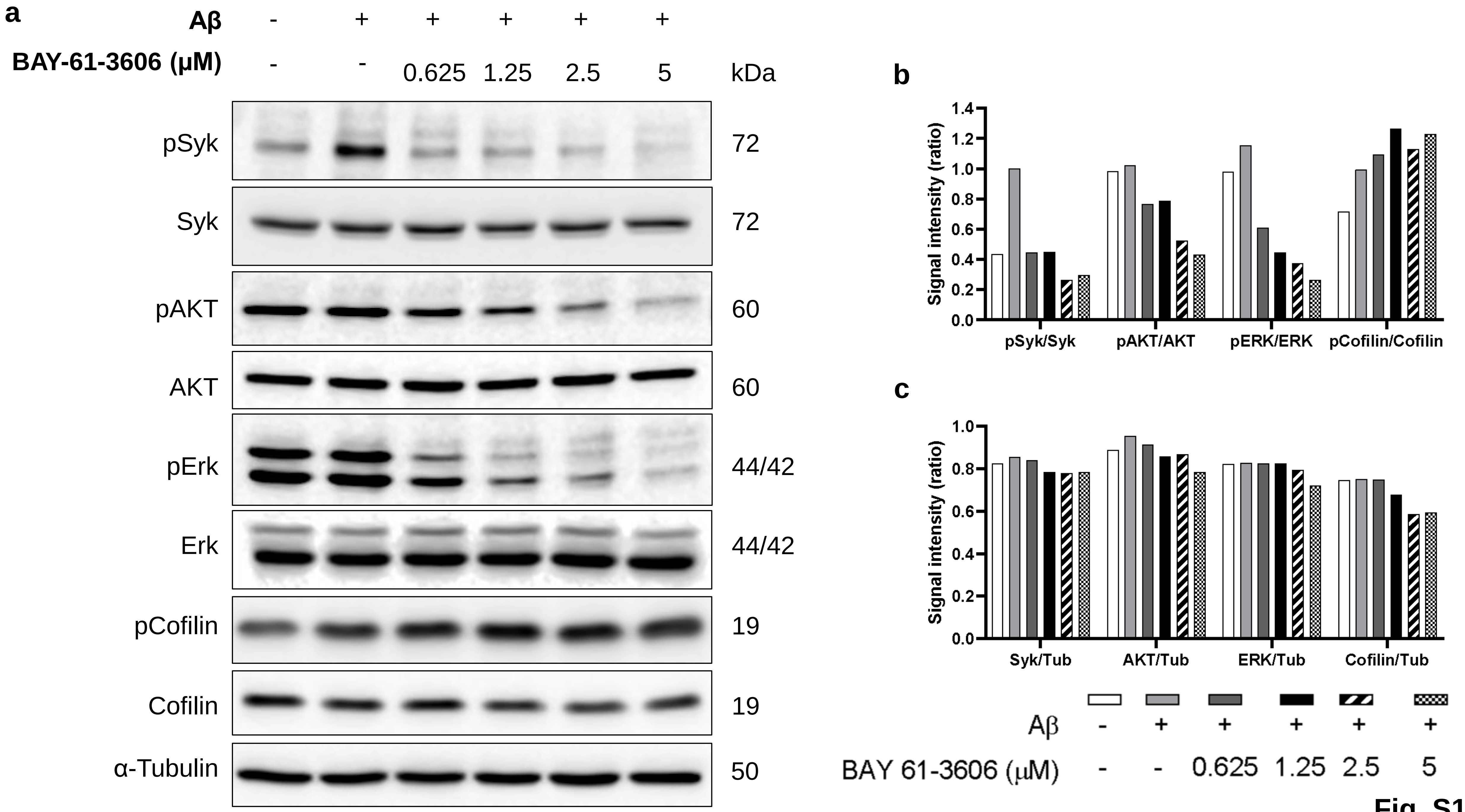
a
Aβ - + + + + +
BAY-61-3606 (μM) - - 0.625 1.25 2.5 5
kDa
pSyk 72
Syk 72
pAKT 60
AKT 60
pErk 44/42
Erk 44/42
pCofilin 19
Cofilin 19
α-Tubulin 50
b
Signal intensity (ratio)
pSyk/Syk
pAKT/AKT
pERK/ERK
pCofilin/Cofilin
c
Signal intensity (ratio)
Syk/Tub
AKT/Tub
ERK/Tub
Cofilin/Tub
Aβ - + + + + +
BAY 61-3606 (μM) - - 0.625 1.25 2.5 5

Fig. S10

Figure S10. Dose-dependent Syk inhibition by BAY-61-3606 in the N9 microglial cell line. BAY-61-3606 was applied to N9 cells at different concentrations, ranging from 0.75 to 5μM for 2h, followed by the addition of aggregated human $A\beta_{42}$ peptide at a concentration of 750nM. (a-c) Western blotting of phospho- and total Syk and downstream signaling molecules from the FcR mediated phagocytosis pathway: AKT, ERK, and Cofilin.

Supplementary Materials and Methods

Vaccine administration. Mice were injected intramuscularly with 25µg DNA (50µl) and electroporation was administered immediately to the area of the injection using a two-needle array electrode, 10mm (BTX, 45-0167, Holliston, MA) and an ECM830 electroporator (BTX, 45-2052). Electroporation configuration: one pulse of 450V/cm, 2 repetitions, duration=0.05ms, interval=0.125s following a second pulse of 110V/cm, 8 repetitions, duration=10ms, interval=0.125s[1].

Serum collection. Blood was extracted from the facial vein using a glass cannula and incubated for 30min at RT to clot. Samples were then centrifuged at 1500×g for 8min at 4°C and clear serum was stored at -20°C for further analysis.

Antibody titer. Anti-$A\beta_{1-11}$ Ab production was quantified by a standard indirect ELISA. 96-well high binding microplates (Microlon, 655061, Greiner bio-one, Monroe, NC) were covered with 50µl of recombinant mouse $A\beta_{1-11}$ peptide (custom synthesis, Adar-Biotech, Yavne, Israel) in carbonate/bicarbonate coating buffer (pH 9.6) at concentration of 3µg/ml. Plates were incubated overnight at 4°C, washed 3 times with 0.1% PBS-Triton and blocked with 2% Bovine Serum Albumin (BSA, A7906, Sigma, St. Louis, MO) for 1h at RT. Plates were washed 3 times with PBS-T following serum incubation at dilutions of 1:100-1:12,500 for 1h at RT. Standard curve was carried out using known concentrations of primary rabbit anti-Aβ Aβ1-14 Ab (50-500ng/ml, ab2539, Abcam, Cambridge, UK). Plates were washed 3 time in PBS-T and incubated with HRP-conjugated goat anti-mouse IgG secondary Abs diluted at 1:5,000, (115-035-003, Peroxidase

AffiniPure, Jackson Immunoresearch, PA) or goat anti-rabbit secondary Ab for standard curve wells (111-035-003, Peroxidase AffiniPure, Jackson Immunoresearch) for 1h at RT. Plates were washed 3 times in PBS-T and 3,3',5,5'-tetramethylbenzidine (TMB) substrate (00-4201-56, Affimetrix eBioscience, San Diego, CA) was applied. Colorimetric reaction was stopped by adding 50μl of 2M H_2SO_4 solution (339741, Sigma-Aldrich, St. Louis, MO). OD was measured at 450nm using a spectrophotometer.

Immunoglobulin isotyping. IgG isotyping was conducted using a similar indirect ELISA protocol with the addition of specific anti-mouse-immunoglobulin Abs (ISO-2, Sigma-Aldrich, St. Louis, MO) diluted at 1:1000, incubated for 30min at RT. Next, donkey anti-goat secondary Ab (705-035-003, Peroxidase AffiniPure Donkey Anti-Goat IgG, Jackson Immunoresearch) diluted at 1:5,000 was applied for 1h at RT.

T-maze. We utilized a variant of the T-maze alternation test modified from[2]. Briefly, T-maze arms were 30cm long and 15cm wide, walls were 15cm high, covered by different black and white patterns. Mice were given 3 trials with a 2hrs inter-trial interval. Each trial consisted of 2 stages: During acquisition, mice were released from the starting chamber and were given the opportunity to enter one of the target arms. A trial was ended when the animals spent more than 2s with all 4 limbs inside one of the target arms. Next, mice were allowed to stay in the chosen arm for 30s, followed by a repetitive trial in which alternation rate was measured.

Measuring A$\beta_{40/42}$ levels using sELISA. Aβ_{40} and Aβ_{42} in the cortex were measured using a modification of a previously published sandwich-ELISA protocol[3]. Briefly, tissues were

mechanically homogenized in in TBS-Triton 1% (120mM NaCl, 50mM Tris, pH=8.0, 150mg/ml, tissue/buffer) including protease inhibitor cocktail (1:100, P2714, Sigma, St. Louis, MO), then incubated on ice for 30min followed by centrifugation for 120min at 17,000g at 4 °C. Supernatant, containing TBS-T-soluble fraction of $A\beta_{40}$ and $A\beta_{42}$ was removed and stored at−20°C. The centrifuged pellet was incubated for 30min with 2% TBS-SDS (120mM NaCl, 50mM Tris + 2% SDS) on ice, following centrifugation at the same conditions as mentioned above. Supernatant, containing the SDS-soluble fraction of Aβ was removed and stored at −20°C. The remaining pellet, containing insoluble Aβ was resuspended in 70% formic acid and incubated on ice for 30m, followed by centrifugation at the same conditions as mentioned above. Formic acid-soluble supernatant was separated and neutralized using 1M Tris (pH=11, 20-time the volume of the formic acid) and stored at −20°C. Total protein concentration was determined using the BCA method (Cat#23225, Thermo Fisher Scientific, Waltham, MA). For the ELISA assay, 96-well polystyrene microplates (655061, Greinerbio-one, Monroe, NC) were covered with 50μl of anti-rabbit-N-terminus $A\beta_{1\text{-}14}$ (ab2539, Abcam, Cambridge, UK) at a concentration of 5μg/ml in carbonate-bicarbonate buffer (pH=9.6) and incubated overnight at 4°C. Plates were washed 4 times in PBS-T solution (0.1% Triton-x in PBS) and blocked with 2% BSA solution in PBS. 50μl of tissue homogenate were applied to each well, and incubated for 60min at RT. Plates were then washed 5 times in PBS-T, and the following detection Abs were added: Anti-$A\beta_{40}$ Ab (ab20068, Abcam, Cambridge, UK) diluted at 1:500 or anti $A\beta_{42}$ Ab (05-831, Millipore, Billerica, MA) at 1:2500, and incubated for 60 min at RT. Next, plates were washed 5 times in PBS-T and secondary goat-anti-mouse IgG HRP-conjugated Ab was added (115-035-003, Peroxidase AffiniPure Goat Anti-Mouse, Jackson immunoresearch, PA) at a dilution of 1:5,000. Plates were washed 5 times with PBS-T and 50μl of 3, 3', 5, 5'-tetramethylbenzidine (TMB) substrate (00-4201-56, Affimetrix

eBioscience, San Diego, CA) was added. The color reaction was allowed to develop for 3 min and was stopped by adding 50µl of 2M H2SO4. Optical density (OD) was measured at 450nm using a spectrophotometer. Standard curve was carried out using known concentrations of recombinant $A\beta_{40}$ and $A\beta_{42}$.

RT-qPCR. Total RNA was extracted using TRIzol Reagent (Ambion, Life Technologies, CA). Complementary DNA (cDNA) was generated using Revert Aid H minus first strand cDNA synthesis kit (Thermo Scientific, Waltham, MA). RT-PCR reactions were performed using Fast SYBR Green Master Mix (Applied Biosystems, CA) in a StepOnePlus instrument (Applied Biosystems, CA). Primers (Supplementary table 2) were calibrated, a negative control was performed for each primer pair and PCR products were validated in gel electrophoresis. Samples were measured in triplicates and values were normalized according to mRNA levels of β-Actin. Denaturation was performed at 94°C for 30s, annealing for 10s and elongation was performed at 72°C for 10s.

Western blot. Hippocampal protein lysate was obtained as mentioned above with the addition of phosphatase inhibitor cocktail (1:100, 524625, Merck-Millipore, Billerica, MA). 25µg per of total protein per sample was boiled in Laemmli buffer at 95°C for 10min and loaded to 10% (w/v) Tris-glycine polyacrylamide gels. Electrophoresed samples were transferred to a PDVF (IPVH00010 Immobilon-P Membrane, PVDF, 0.45µm, Merck, Kenilworth, NJ) and blocked for unspecific binding using 5% BSA (A7906, Sigma, St. Louis, MO) for phospho-proteins and 5% skim milk (M530, Himedia, Mumbai, India) for unphosphorylated proteins, diluted in 0.1% (v/v) PBS-Tween20 (0.1% Tween 20, P9416-50ML, Sigma, St. Louis, MO). Next, membranes were

incubated with a primary Ab overnight at 4°C. Full Abs details can be found in Supplementary table 1. Unbound Abs were washed 3 times in PBS-T for 5min followed by membrane incubation with HRP-conjugated goat anti mouse IgG secondary Ab (Cat#115-035-003, Peroxidase AffiniPure, Jackson immunoresearch) or goat anti-rabbit secondary Ab (111-035-003, Peroxidase AffiniPure, Jackson Immunoresearch) diluted at 1:10,000 in blocking buffer for 1h at room temperature (RT). Ab-Ag bindings were detected by applying ECL (ECL kit, 20-500-120, Biological industries, Israel).

Supplementary references.

1 Olkhanud, P. B. *et al.* DNA immunization with HBsAg-based particles expressing a B cell epitope of amyloid beta-peptide attenuates disease progression and prolongs survival in a mouse model of Alzheimer's disease. *Vaccine* **30**, 1650-1658, doi:10.1016/j.vaccine.2011.12.136 (2012).

2 Deacon, R. M. & Rawlins, J. N. T-maze alternation in the rodent. *Nature protocols* **1**, 7-12, doi:10.1038/nprot.2006.2 (2006).

3 Illouz, T., Madar, R., Griffioen, K. & Okun, E. A protocol for quantitative analysis of murine and human amyloid-beta1-40 and 1-42. *J Neurosci Methods* **291**, 28-35, doi:10.1016/j.jneumeth.2017.07.022 (2017).

Supplementary Table 1. sELISA, IF and WB primary antibodies

Protein	Application	Catalog #	Manufacturer	Dilution	Host	Secondary Ab	Manufacturer	Dilution
$A\beta_{1-14}$	sELISA (capture)	ab2539	Abcam, Cambridge, UK	1:200	Rabbit	N/A		
$A\beta_{1-40}$	sELISA (detection)	ab20068	Abcam, Cambridge, UK	1:500	Mouse	Anti-mouse IgG, HRP	115-035-003, Jackson immunoresearch	1:5000
$A\beta_{1-42}$	sELISA (detection)	05-381-I	Millipore, Billerica, MA	1:2000	Mouse	Anti-mouse IgG, HRP	115-035-003, Jackson immunoresearch	1:5000
$A\beta_{1-42}$	IF	05-381-I	Millipore, Billerica, MA	1:1000	Mouse	Anti-mouse IgG, Alexa-488/568	Invitrogen	1:1000
Iba1	IF	019-19741	Wako, Osaka, Japan	1:1000	Rabbit	Anti-rabbit IgG, Alexa-488/568/647	Invitrogen	1:1000
NeuN	IF	MAB377	Millipore, Billerica, MA	1:10000	Mouse	Anti-mouse IgG, Alexa-488/568	Invitrogen	1:1000
GFAP	IF	M0761	Agilent, Santa-Clara, CA	1:7500	Rabbit	Anti-rabbit IgG, Alexa-488/568/647	Invitrogen	1:1000
FcγRI	IF	MCA5997	Bio-Rad, Hercules, CA	1:1000	Rat	Anti-rat IgG, Alexa-488/568	Invitrogen	1:1000
FcγRIIb	IF	MCA6001	Bio-Rad, Hercules, CA	1:200	Rat	Anti-rat IgG, Alexa-488/568	Invitrogen	1:1000

FcγRIII	IF	MCA5998	Bio-Rad, Hercules, CA	1:1000	Rat	Anti-rat IgG, Alexa-488/568	Invitrogen	1:1000
FcγRIV	IF	MCA5999	Bio-Rad, Hercules, CA	1:1000	Rat	Anti-rat IgG, Alexa-488/568	Invitrogen	1:1000
CD68	IF	ab53444	Abcam, Cambridge, UK	1:2250	Rat	Anti-rat IgG, Alexa-488/568	Invitrogen	1:1000
pSyk	IF	CST-2710	Cell-Signaling, Danvers, MA	1:500	Rabbit	Anti-rabbit IgG, Alexa-488/568/647	Invitrogen	1:1000
pSyk	WB	CST-2710	Cell-Signaling, Danvers, MA	1:1000	Rabbit	Anti-rabbit IgG, HRP	111-035-003, Jackson Immunoresearch	1:10000
Syk	WB	CST-13198	Cell-Signaling, Danvers, MA	1:1000	Rabbit	Anti-rabbit IgG, HRP	111-035-003, Jackson Immunoresearch	1:10000
pAKT	WB	CST-4060	Cell-Signaling, Danvers, MA	1:2000	Rabbit	Anti-rabbit IgG, HRP	111-035-003, Jackson Immunoresearch	1:10000
AKT	WB	CST-2920	Cell-Signaling, Danvers, MA	1:2000	Mouse	Anti-mouse IgG, HRP	115-035-003, Jackson immunoresearch	1:10000
pERK	WB	CST-4370	Cell-Signaling, Danvers, MA	1:2000	Rabbit	Anti-rabbit IgG, HRP	111-035-003, Jackson Immunoresearch	1:10000
ERK	WB	CST-4696	Cell-Signaling, Danvers, MA	1:2000	Mouse	Anti-mouse IgG, HRP	115-035-003, Jackson immunoresearch	1:10000
β-Tubulin	WB	T5076	Sigma-Aldrich, St. Louis, MO	1:10000	Mouse	Anti-mouse IgG, HRP	115-035-003, Jackson immunoresearch	1:10000

pCofilin	WB	CST-3313	Cell-Signaling, Danvers, MA	1:1000	Rabbit	Anti-rabbit IgG, HRP	111-035-003, Jackson Immunoresearch	1:10000
Cofilin	WB	ab54532	Abcam, Cambridge, UK	1:500	Mouse	Anti-mouse IgG, HRP	115-035-003, Jackson immunoresearch	1:10000
β-Actin	WB	sc-47778	Santa-Cruz Biotechnology, Dallas, TX	1:1000	Mouse	Anti-mouse IgG, HRP	115-035-003, Jackson immunoresearch	1:10000
hAPP	WB	803001	Biolegend, San Diego, CA	1:5,000	Mouse	Anti-mouse IgG, HRP	115-035-003, Jackson immunoresearch	1:10000

Supplementary Table 2. RT-PCR primers

Gene	Forward primer	Reverse primer	Annealing temp (°C)	Product size (bp)
FCGR1	AGGTTCCTCAATGCCAAGTG	ATTCTTCCATCCGTGACACC	60	127
FCGR3	TATCGGTGTCAAATGGAGCA	TATGGCACCTTAGCGTGATG	60	130
FCGR4	CGAGGACAATTCTATCAAGTGGTT	ACTTAGTGGTCTGAAGCAATAGCC	60	188
FCRN	ACTGCTAGGCCACCTGGAG	AGGAGAAAGCAGCACAGGTC	64	122
CD68	ACTTCGGGCCATGTTTCTCT	GCTGGTAGGTTGATTGTCGT	60	138
ACTIN	TTCTTTGCAGCTCCTTCGTT	ATGGAGGGGAATACAGCCC	56	149
TYROBP	GGTGTTGACTCTGCTGATTGC	AAGCTCCTGATAAGGCGACTC	56	127
