# Supplementary material for: Maternal antibodies facilitate Amyloid-β clearance by activating Fc-receptor-Syk-mediated phagocytosis
Source: Commun Biol. 2021 Mar 12;4:329. doi: 10.1038/s42003-021-01851-6 (PMC7955073; doi:10.1038/s42003-021-01851-6)
Supplement: Supplementary file 2 — Reporting Summary [file 42003_2021_1851_MOESM2_ESM.pdf]

nature research

Corresponding author(s): Tomer Illouz

Last updated by author(s): Jan 25, 2021

Reporting Summary

Nature Research wishes to improve the reproducibility of the work that we publish. This form provides structure for consistency and transparency in reporting. For further information on Nature Research policies, see our Editorial Policies and the Editorial Policy Checklist.

Statistics

For all statistical analyses, confirm that the following items are present in the figure legend, table legend, main text, or Methods section.

n/a	Confirmed	
☐	☒	The exact sample size (*n*) for each experimental group/condition, given as a discrete number and unit of measurement
☐	☒	A statement on whether measurements were taken from distinct samples or whether the same sample was measured repeatedly
☐	☒	The statistical test(s) used AND whether they are one- or two-sided<br>*Only common tests should be described solely by name; describe more complex techniques in the Methods section.*
☐	☒	A description of all covariates tested
☐	☒	A description of any assumptions or corrections, such as tests of normality and adjustment for multiple comparisons
☐	☒	A full description of the statistical parameters including central tendency (e.g. means) or other basic estimates (e.g. regression coefficient) AND variation (e.g. standard deviation) or associated estimates of uncertainty (e.g. confidence intervals)
☒	☐	For null hypothesis testing, the test statistic (e.g. *F*, *t*, *r*) with confidence intervals, effect sizes, degrees of freedom and *P* value noted<br>*Give P values as exact values whenever suitable.*
☒	☐	For Bayesian analysis, information on the choice of priors and Markov chain Monte Carlo settings
☒	☐	For hierarchical and complex designs, identification of the appropriate level for tests and full reporting of outcomes
☒	☐	Estimates of effect sizes (e.g. Cohen's *d*, Pearson's *r*), indicating how they were calculated

Our web collection on statistics for biologists contains articles on many of the points above.

Software and code

Policy information about availability of computer code

Data collection	n/a
Data analysis	Plaque and microglia quantification were conducted using the MATLAB 2017a blob detection function

For manuscripts utilizing custom algorithms or software that are central to the research but not yet described in published literature, software must be made available to editors and reviewers. We strongly encourage code deposition in a community repository (e.g. GitHub). See the Nature Research guidelines for submitting code & software for further information.

Data

Policy information about availability of data

All manuscripts must include a data availability statement. This statement should provide the following information, where applicable:

- Accession codes, unique identifiers, or web links for publicly available datasets
- A list of figures that have associated raw data
- A description of any restrictions on data availability

Source data behind the graphs are is available on the Open Science Framework: https://osf.io/jnxrz/. All the data supporting the findings of this study are freely available upon request.

Field-specific reporting

Life sciences study design

All studies must disclose on these points even when the disclosure is negative.

Sample size	Sample size was determined according to power analysis using the G-power tool, for effect size of 0.8 and P-value of <5%
Data exclusions	Outliers were identified using the robust regression and outlier removal (ROUT) method with coefficient Q=1%
Replication	Immunization experiments in dams were replicated in 10-12 animals per group.<br>Maternal/active immunization were replicated in 5 animals per group.<br>Antibody titer, qPCR and Amyloid sELISA were conducted in triplicates.<br>Plaque histology was conducted with 10 brain slices per animal<br>in vitro studies were conducted with 3 replicates per condition
Randomization	Naive dams were randomly allocated to control/vaccine groups. Maternally/control vaccinated offspring were not randomly allocated to control/active vaccination to maintain original littermates at the same cages.
Blinding	All behavioral testing were conducted under experimenter blinding to group allocation

Reporting for specific materials, systems and methods

We require information from authors about some types of materials, experimental systems and methods used in many studies. Here, indicate whether each material, system or method listed is relevant to your study. If you are not sure if a list item applies to your research, read the appropriate section before selecting a response.

Materials & experimental systems

n/a	Involved in the study	
☐	☒	Antibodies
☐	☒	Eukaryotic cell lines
☒	☐	Palaeontology and archaeology
☐	☒	Animals and other organisms
☒	☐	Human research participants
☒	☐	Clinical data
☒	☐	Dual use research of concern

Methods

n/a	Involved in the study	
☒	☐	ChIP-seq
☐	☒	Flow cytometry
☒	☐	MRI-based neuroimaging

Antibodies

Antibodies used

Rabbit anti-Aβ1-14 [sELISA (capture)] ab2539 Abcam, Cambridge, UK 1:200
Mouse anti-Aβ1-40 [sELISA (detection)] ab20068 Abcam, Cambridge, UK 1:500
Goat anti-mouse IgG, HRP [sELISA, ELISA] 115-035-003, Jackson immunoresearch 1:5000
Mouse anti-Aβ1-42 sELISA [sELISA (detection)] 05-381-I Millipore, Billerica, MA 1:2000
Mouse anti-Aβ1-42 [IF] 05-381-I Millipore, Billerica, MA 1:1000
Goat anti-mouse IgG, Alexa-488/568 Invitrogen 1:1000
Rabbit anti-Iba1 [IF] 019-19741 Wako, Osaka, Japan 1:1000
Goat anti-rabbit IgG, Alexa-488/568/647 Invitrogen 1:1000
Mouse anti-NeuN [IF] MAB377 Millipore, Billerica, MA 1:10000
Rabbit anti-GFAP [IF] M0761 Agilent, Santa-Clara, CA 1:7500 Rabbit
Rat anti-FcγRI [IF] MCA5997 Bio-Rad, Hercules, CA 1:1000
Goat anti-rat IgG, Alexa-488/568 Invitrogen 1:1000
Rat anti-FcγRIIb [IF] MCA6001 Bio-Rad, Hercules, CA 1:200
Rat anti FcγRIII [IF] MCA5998 Bio-Rad, Hercules, CA 1:1000
Rat anti FcγRIV [IF] MCA5999 Bio-Rad, Hercules, CA 1:1000
Rant anti-CD68 [IF] ab53444 Abcam, Cambridge, UK 1:2250
Rabbit anti-pSyk [IF] CST-2710 Cell-Signaling, Danvers, MA 1:500
Goat anti-rabbit IgG, HRP 111-035-003, Jackson Immunoresearch 1:10000
Rabbit anti-Syk [WB] CST-13198 Cell-Signaling, Danvers, MA 1:1000 A
Rabbit anti-pAKT [WB] CST-4060 Cell-Signaling, Danvers, MA 1:2000
Mouse anti-AKT [WB] CST-2920 Cell-Signaling, Danvers, MA 1:2000
Rabbit anti-pERK [WB] CST-4370 Cell-Signaling, Danvers, MA 1:2000
Mouse anti-ERK [WB] CST-4696 Cell-Signaling, Danvers, MA 1:2000
Mouse anti-β-Tubulin [WB] T5076 Sigma-Aldrich, St. Louis, MO 1:10000
Rabbit anti-pCofilin [WB] CST-3313 Cell-Signaling, Danvers, MA 1:1000
Mouse anti-Cofilin [WB] ab54532 Abcam, Cambridge, UK 1:500

	Mouse anti-β-Actin [WB] sc-47778 Santa-Cruz Biotechnology, Dallas, TX 1:1000<br>Mouse anti-hAPP (6E10) [WB] 803001 Biolegend, San Diego, CA 1:5,000 Mouse
Validation	- ELISA antibodies were validated by testing cross-reactivity between human/mouse antigens<br>- sELISA antibodies were validated in our previous publication (see methods section)<br>- Fcgr antibodies were pre-validated using staining of spleen<br>- All WB antibodies were pre-validated using blotting of brain protein homogenates from adult transgenic and WT brains

Eukaryotic cell lines

Policy information about cell lines

Cell line source(s)	N9 mouse embryonic microglia cell line, provided by the Weitzmann Institute of Science, Rechovot, Israel
Authentication	Cells were authenticated by testing positive for microglia markers and negative staining for astrocytic or neuronal markers
Mycoplasma contamination	Cell lines were not tested for mycoplasma contamination
Commonly misidentified lines<br>(See ICLAC register)	n/a

Animals and other organisms

Policy information about studies involving animals; ARRIVE guidelines recommended for reporting animal research

Laboratory animals	The C57BL strain (The C57BL strain (Jackson Laboratories #000664) was used as WT control.<br>The 5XFAD (Jackson Laboratories #34840) model of early-onset Alzheimer's disease was used to model early Amyloid-b accumulation
Wild animals	No wild animals were used.
Field-collected samples	No field-collected samples were taken.
Ethics oversight	Animal care and experimental procedures followed the NIH Guide for the Care and Use of Laboratory Animals and were approved by the Bar-Ilan University Animal Care and Use Committee

Note that full information on the approval of the study protocol must also be provided in the manuscript.

Flow Cytometry

Plots

Confirm that:

- [x] The axis labels state the marker and fluorochrome used (e.g. CD4-FITC).
- [x] The axis scales are clearly visible. Include numbers along axes only for bottom left plot of group (a 'group' is an analysis of identical markers).
- [ ] All plots are contour plots with outliers or pseudocolor plots.
- [x] A numerical value for number of cells or percentage (with statistics) is provided.

Methodology

Sample preparation	Murine embryonic microglia cell-line N9 were grown in Dulbecco's modified Eagle's medium (DMEM), supplemented with 10% fetal bovine serum (FBS), penicillin, streptomycin, and L-glutamine for 3-4 days to reach confluence. For beads phagocytosis assay, N9 cells were deprived of FBS and incubated this Syk inhibitor BAY-61-3606 (5µM) for 3h. 1µm-fluorescent beads (L1030, Sigma, St. Louis, MO) were pre-coated with FBS for 1h at 37°C, then applied to cells at a dilution of 1:1000 for 1h at 37°C. Cells were then washed five times with PBS (containing calcium and magnesium) and trypsinized. Phagocytic FITC+ cell count was conducted using the BD-LSRFortessa cell analyzer (BD bioscience, East Rutherford, NJ). For Aβ phagocytosis assay, cells were fixed with 4% PFA, permeabilized using 0.3% PBS-triton, and stained for intracellular Aβ using an anti-Aβ1-14 antibody.
Instrument	BD-LSRFortessa cell analyzer (BD bioscience, East Rutherford, NJ)
Software	Data was collected using the BD FACSDiva™ Software. Data was amalyzed using the FlowJo software v10.6.1
Cell population abundance	FACS analysis was conducted on N9 cell line only, assumed to be 100% pure
Gating strategy	Cell debris, apoptotic cells and cell doublets were excluded in the preliminary FSC-A/SSC-A gates, as these population are distinct from the live, single-cell population. Single cells were confirmed by FSC-A/FSC-H linearity. Negative staining was determined according to Naive cells in the FBS-beads experiment and unstained sample in the Amyloid-b experiment. Both gates are depicted in Figure 6.B,G

- [ ] Tick this box to confirm that a figure exemplifying the gating strategy is provided in the Supplementary Information.
